# Cerebral palsy in a total population of 4–11 year olds in southern Sweden. Prevalence and distribution according to different CP classification systems

**DOI:** 10.1186/1471-2431-7-41

**Published:** 2007-12-05

**Authors:** Lena Westbom, Gunnar Hagglund, Eva Nordmark

**Affiliations:** 1 Division of Paediatrics, Department of Clinical Sciences (Lund), Lund University, Lund, Sweden; 2 Department of Orthopaedics, Department of Clinical Sciences (Lund), Lund University, Lund, Sweden; 3 Division of Physiotherapy, Department of Health Sciences, Lund University, Lund, Sweden; 4 Children's Hospital, University Hospital, SE 221 85 Lund, Sweden

## Abstract

**Background:**

The aim of this study was to investigate the prevalence of cerebral palsy (CP) as well as to characterize the CP population, its participation in a secondary prevention programme (CPUP) and to validate the CPUP database.

**Methods:**

The study population was born 1990–1997 and resident in Skåne/Blekinge on Jan 1^st ^2002. Multiple sources were used. Irrespective of earlier diagnoses, neuropaediatrician and other professional medical records were evaluated for all children at the child habilitation units. The CPUP database and diagnosis registers at hospital departments were searched for children with CP or psychomotor retardation, whose records were then evaluated. To enhance early prevention, CP/probable CP was searched for also in children below four years of age born 1998–2001.

**Results:**

The prevalence of CP was 2.4/1,000 (95% CI 2.1–2.6) in children 4–11 years of age born in Sweden, excluding post-neonatally acquired CP. Children born abroad had a higher prevalence of CP with more severe functional limitations. In the total population, the prevalence of CP was 2.7/1,000 (95% CI 2.4–3.0) and 48% were GMFCS-level I (the mildest limitation of gross motor function).

One third of the children with CP, who were born or had moved into the area after a previous study in 1998, were not in the CPUP database. The subtype classification in the CPUP database was adjusted in the case of every fifth child aged 4–7 years not previously reviewed.

**Conclusion:**

The prevalence of CP and the subtype distribution did not differ from that reported in other studies, although the proportion of mild CP tended to be higher.

The availability of a second opinion about the classification of CP/CP subtypes is necessary in order to keep a CP register valid, as well as an active search for undiagnosed CP among children with other impairments.

## Background

Primary prevention of cerebral palsy (CP) through improvements in maternal health, perinatal care and accident prevention is desirable. CP registers are usually created to examine aetiological factors, especially the outcome of perinatal care [1-8]. The CP inventory results presented in this study is connected to a secondary prevention programme. Prevention of hip dislocation and severe contractures and deformities is the main priority of the follow-up programme, entitled CPUP, for children with CP. It has been ongoing in the southernmost part of Sweden (Skåne/Blekinge) since 1994. Other aims of the programme are to describe the "natural" course of functioning and development in CP, evaluate treatment methods and increase cooperation between health care professionals. The CPUP programme has been effective in Skåne/Blekinge [9-11] and the CPUP database was approved as a national health care quality register by the Swedish National Board of Health and Welfare in 2005. At present, the whole of Sweden as well as areas encompassing half of the Norwegian population participate.

Inventories among children living in the Skåne/Blekinge area are performed regularly in order to offer all children with CP the opportunity to participate in the CPUP programme. The CP diagnosis, subtype and motor function of the child are ascertained and the CPUP database corrected when necessary. Two studies of the previous inventory described all children aged 4–7 years with CP who were living in the area in 1998 [12,13]. The aims of the present study were to up-date the 1998 CP-register, to investigate the prevalence of CP as well as to characterise the 4–11 year old CP population and its participation in the follow-up programme. An additional aim was to determine whether reports from the neuropaediatricians and therapists responsible for the child's treatment are sufficient to keep the CPUP database complete and valid.

## Methods

The study area, the counties of Skåne and Blekinge, comprised a total population of 1.3 million on Jan 1^st ^2002. The geographical area was divided into 13 child habilitation districts that offered services to about two percent of the child population with a range of developmental disabilities. Neuropaediatricians, physio- (PTs) and occupational therapists (OTs) collaborate and offer all children with probable or defined CP participation in the CPUP at the earliest possible stage. The CPUP database is continuously updated by the local habilitation teams. Twice a year before the age of six and once a year thereafter, the local PTs and OTs document the actual functioning of the child. Children who do not fulfil the CP criteria at 4–7 years of age leave the programme and are removed from the CPUP database.

Multiple sources of information about the child population were used during the study: the Swedish population register, the 1998 CP- register, the CPUP database, diagnosis registers in hospitals/paediatric clinics as well as those from habilitation centres. In order to locate every child with CP in the area, all medical records at the habilitation units were studied by the authors (EN, LW), irrespective of diagnosis. Files from the paediatric hospital departments/clinics were scrutinized for children with registered diagnoses CP or psychomotor retardation. Descriptions by doctors and therapists were studied in order to identify CP and the subtype. The history, body structure/function, including neurological status, capability and performance of gross- and fine motor activities were evaluated for every child. Data on motor function and spasticity/muscle tone were available from the CPUP database for those followed by the programme. When necessary, efforts were made to determine the diagnosis and the CP subtype by means of videos and discussions with the responsible doctor and therapists, or the child was assessed by the author LW.

The inventory comprised a search for CP/probable CP in children less than four years of age, born 1998–2001, as well as a classification of the CP diagnosis and subtypes in children born 1994–97, aged 4–7 years. In the 8–11 year cohort, born 1990–93, a re-evaluation was performed including those who had moved into the area after 1998. Information was collected about the health and functioning of the children ascertained as close as possible to Jan 1^st ^2002.

Cerebral palsy was defined according to Mutch *et al.*[14] with a cut-off for post-neonatal causes of CP from day 29 to before the second birthday. Other exclusion and inclusion criteria were in accordance with the Surveillance of Cerebral Palsy in Europe (SCPE) definition of CP [3]. Also children with motor impairment and specific neurological signs (ataxia, dyskinesia and/or spasticity) caused by different genetic syndromes without progressive brain dysfunction were included.

The CP subtypes were classified according to the Swedish (SC) and the European (SCPE) classifications [1,3,15,16]. The SC, which was applied from the start of the CPUP programme, includes three spastic syndromes: spastic hemiplegia, spastic diplegia and spastic tetraplegia. Spastic tetraplegia is defined as massive total motor disability involving all four limbs, the upper to at least the same degree as the lower ones. All cases in which the lower limbs are more affected than the upper ones are assigned to the spastic diplegic CP group. The ataxic forms comprise ataxic diplegia and simple ataxia including dysequilibrium syndrome [17]. The dyskinetic subgroups are choreo-athetosis and tonus-changing (dystonia) and also include a third transitional group, dystonia plus choreo-athetosis, in which neither dyskinetic sign is dominant. A new CP subtype classification was introduced by the SCPE network in 2000 [3]. In the SCPE decision tree, the spastic subtypes are unilateral (USCP) and bilateral spastic CP (BSCP). Dyskinetic CP is divided into a tonus-changing (dystonic) and a choreo-athetotic type. According to the SCPE classification dystonic CP is characterised by hypokinesia as well as a tendency to increased muscle tone, while hyperkinesia and a tendency to decreased muscle tone are typical of the choreo-athetotic subtype. Both subtype classifications (SCPE and SC) are based on the dominant neurological sign, although they include a mixed (SC) or non-classifiable (SCPE) category for exceptional cases. In this study the CP subtype was classified at 4–7 years of age. In the 8–11 year olds, the classification was based on their status at 4–7 years in the 1998 inventory [12].

Gross motor function was determined according to the Gross Motor Function Classification System (GMFCS) with focus on the child's current self-initiated movements, in particular sitting and walking [18]. The reliability, validity and stability over time have been established [18-21]. The GMFCS comprises five levels, each of which is characterized by a description of the gross motor function in four age intervals (<2, 2–<4, 4–<6 and 6–<12 years). Children in GMFCS level I can perform all gross motor activities of their age-matched peers, albeit with some difficulty in terms of speed, balance and coordination. Children in level V have difficulty controlling their head and trunk posture in most positions, and voluntary control of movement is severely limited. The GMFCS-level used was that established and reported by the child's regular physiotherapist as close as possible to Jan 1^st ^2002.

The point prevalence was calculated as the number of children with CP living in the counties of Skåne and Blekinge on Jan 1^st ^2002 in relation to the total number of children of the same age, place of birth and gender living in the area at that time based on data from Statistics Sweden [22]. The population under study consisted of 70,090 children born between 1990 and 1993 and 55,474 born between 1994 and 1997, of which 4,584 and 2,290 respectively were born abroad. The binomial distribution exact method with 95% confidence intervals (95% CI) of the prevalence rates are presented.

The study was approved by the Medical Research Ethics Committee at Lund University (LU-443-99).

## Results

### Prevalence and distribution of CP according to gender, age, place of birth, GMFCS in relation to the different CP subtypes

The prevalence of CP in children aged between 4 and 11 was 2.7/1,000 (95% CI 2.4–3.0). Children born outside Sweden had a higher prevalence of CP, 6.7/1,000 (95% CI 4.8–8.8), than those born in Sweden. Excluding post-neonatally acquired CP and children born abroad, the prevalence was 2.4/1,000 (95% CI 2.1–2.6). The ratio boys:girls was 1.4:1. In Table 1, the prevalence of CP according to age group, gender and place of birth is presented.

**Table 1:** Prevalence of cerebral palsy

	Total population		Born in Sweden		Born outside Sweden	
	Number of children	Prevalence (95% CI)	Number of children	Prevalence (95% CI)	Number of children	Prevalence (95% CI)

Born 1990–93	193	2.8 (2.4 – 3.2)	163	2.5 (2.1 – 2.9)	30	6.5 (4.2 – 8.8)
Born 1994–97	150	2.6 (2.2 – 3.0)	134	2.4 * (2.0 – 2.8)	16	7.0 (3.6 – 10.4)
						
Girls	143	2.3 (1.9 – 2.7)	121	1.9 (1.6 – 2.3)	22	6.6 (3.8 – 9.3)
Boys	200	3.1 (2.6 – 3.4)	176	2.7 (2.3 – 3.1)	24	6.8 (4.1 – 9.5)
						

Total	343	2.7 (2.4 – 3.0)	297	2.5** (2.2 – 2.8)	46	6.7 (4.8 – 8.8)

Cerebral palsy prevalence per 1,000 children born 1990–1997 and resident in the counties of Skåne and Blekinge on Jan 1^st ^2002. CI = confidence interval.

* 2.3/1,000 (95% CI 2.0–2.7) and ** 2.4/1,000 (95% CI 2.1–2.6) excluding post-neonatal aetiology

**Figure 1:**
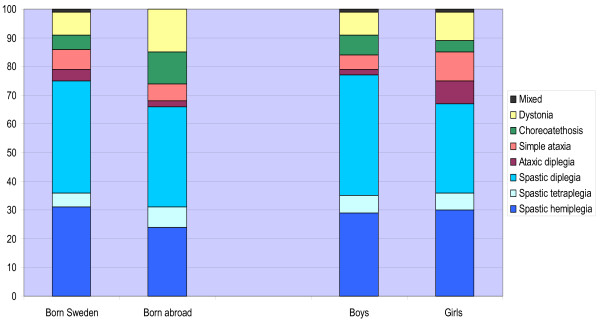
**CP subtypes according to gender and place of birth**. Distribution of CP subtypes (Swedish classification) according to gender and place of birth. Total population of 4–11 year olds (n = 343).

The prevalence and distribution of the CP subtypes are presented in Table 2 and the distribution according to gender and place of birth in Figure 1. Of the SC subtypes, spastic diplegia was more prevalent in boys, 1.3/1,000, than in girls, 0.7/1,000 (95% CI 1.0 – 1.6 and 0.5–0.9/1,000). This was not the case with ataxic diplegia, where the prevalence in girls was 0.18/1,000 and in boys, 0.05/1,000 (95% CI intervals 0.07–0.28 and 0–0.10/1,000).

**Table 2:** Distribution and prevalence of the CP subtypes

SCPE	SC	Children		Prevalence/1,000	Prevalence 95% CI
				
		n	%		
Unilateral spastic	Spastic hemiplegia	104	30	0.8	0.7–1.0
Bilateral spastic	Spastic tetraplegia	19	5.5	0.2	0.1–0.2
	Spastic diplegia	130	38	1.0	0.8–1.2
Ataxic	Ataxic diplegia	14	4	0.1	0.1–0.2
	Simple ataxia	24	7	0.2	0.1–0.3
Choreo-athetosis	Choreo-athetosis	19	5.5	0.2	0.1–0.2
Dystonia	Athetosis + dystonia	5	1.5		
	Dystonia	26	9.5	0.2	0.2–0.3
Non-classifiable	Mixed	2	0.5		

Total		343		2.7	2.4–3.0

Distribution of CP subtypes based on the Surveillance of Cerebral Palsy in Europe (SCPE) and the Swedish (SC) classification systems. Prevalence per 1,000 in children born 1990–1997 and resident in the counties of Skåne and Blekinge on Jan 1^st ^2002. Children born abroad and children with post-neonatally acquired CP included. CI = confidence interval.

The gross motor function ability in the different CP subgroups as laid down in the GMFCS levels is presented in Table 3. Children with the spastic tetraplegia SC subtype had the most severe gross motor disability level, GMFCS V, which level was only found in four percent of the children with spastic diplegia (SC). All five GMFCS levels were common in the BSCP SCPE subgroup, which includes spastic di- and tetraplegia (SC). Twelve out of the 19 children with choreo-athetosis as the dominant neurological sign had mild gross motor dysfunction (GMFCS I-II), but none of the children with dystonia had mild motor dysfunction.

**Table 3:** GMFCS-levels in relation to CP subtypes

SCPE	SC	GMFCS I	GMFCS II	GMFCS III	GMFCS IV	GMFCS V
Unilateral spastic	Spastic hemiplegia	89	12	3	0	0
Bilateral spastic	Spastic tetraplegia	0	0	0	0	19
	Spastic diplegia	54	26	27	18	5
Ataxic	Ataxic diplegia	1	9	4	0	0
	Simple ataxia	13	19	2	0	0
Choreo-athetosis	Choreo-athetosis	8	4	0	6	1
Dystonia	Athetosis + dystonia	0	0	1	1	3
	Dystonia	0	0	4	7	15
Nonclassifiable	Mixed	0	0	0	0	2

Total		165	60	41	32	45

Number of children with different GMFCS-levels in relation to the CP subtypes, based on the Surveillance of Cerebral Palsy in Europe (SCPE) and the Swedish (SC) classification systems. Total population of children born 1990–1997 and resident in the counties of Skåne and Blekinge on Jan 1^st ^2002. Children born abroad and those with post-neonatal CP are included.

The majority (66%) of the children with CP was able to walk independently; almost half of all children with CP (48%) had the highest level of gross motor function, GMFCS level I. The prevalence of CP GMFCS II-V was 1.4/1,000 (95% CI 1.2–1.6/1,000). The GMFCS-specific prevalence rates are shown in Table 4. The prevalence of severe CP, GMFCS levels IV-V, was 1.9/1,000 in children born outside Sweden and 0.5/1,000 in children born in the country (95% CI 0.9–2.9 and 0.4–0.7/1,000).

**Table 4:** Distribution of GMFCS-levels in relation to gender and place of birth

	Children		Prevalence		Boys		Girls		Born in Sweden		Born outside Sweden	
GMFCS	n	%	/1,000	95% CI	n	%	n	%	n	%	n	%
I	165	48	1.3	1.1–1.5	101	51	64	45	146	49	19	41
II	60	18	0.5	0.4–0.6	30	15	30	21	55	19	5	11
III	41	12	0.3	0.2–0.4	25	12	16	11	32	11	9	20
IV	32	9	0.3	0.2-0.3	18	9	14	10	27	9	5	11
V	45	13	0.4	0.3–0.5	26	13	19	13	37	12	8	17

Total	343		2.7	2.4–3.0	200		143		297		46	

Distribution of GMFCS-levels in relation to gender and place of birth; number, percent and prevalence per 1,000 in children born 1990–1997 and resident in the counties of Skåne and Blekinge on Jan 1^st ^2002. Children born abroad and those with post-neonatal CP have been included. CI = confidence interval.

The prevalence of GMFCS I in boys was 1.5/1,000 and in girls 1.0/1,000 (95% CI 1.2–1.8 versus 0.8–1.3/1,000), while the gender difference in the prevalence of GMFCS levels II-V appeared to be less: 1.5/1,000 in boys and 1.3/1,000 in girls (95% CI 1.2–1.8 versus 1.0–1.6/1,000)

### Re-examination of children born 1990–93

The cohort with CP born 1990–93 increased from 167 children in the previous to 193 in the present inventory, although three children had died and one child had moved away from the area; 23 children with CP had moved in, eight of whom were born abroad. Seven children who lived in the area, but who had not been diagnosed as having CP in the previous study, were identified. Four of them had prenatal syndromes that were not included in 1998 in accordance with the guidelines presented by Mutch *et al.*[14].

### CPUP database completeness and validity

According to the inventory results, the 2002 CP-register, six children between 4 and 11 years of age in the CPUP database did not fulfil the criteria for CP and were excluded. Five of the 343 children with CP in this age group declined participation in the programme and were not in the CPUP database.

In the 4–7 year age group (born 1994–97), the CP subtype listed in the CPUP database was changed in 25/131 children (19%). The CPUP database did not include half of the children with CP who had moved into the area after the 1998 inventory, nor one third of the children aged 0–3 years with CP/probable CP. These children were invited to join the follow-up programme.

The total population with CP and their correct CP diagnosis and subtypes established during the inventory study were used in the analysis of CP prevalence and distribution.

## Discussion

This study is not directly comparable to studies on CP incidence and aetiology, due to its focus on prevalence and descriptions of the CP-population as a basis for secondary prevention. It includes a broad age group and children born both in and outside Sweden. In children aged 4–7 years, born in Sweden 1994–97, the prevalence of CP excluding post-neonatal aetiology was 2.3/1,000 (95% CI 2.0–2.7) compared to 2.0/1,000 (95% CI 1.6–2.4) in the cohort born 1990–93 in the 1998 study [12]. The prevalence in children born 1995–98 and resident in western Sweden in December 2002 was 1.92/1,000, and a trend towards a decreasing prevalence compared to the birth years 1991–94 was reported [8].

In addition to the outcome of perinatal care and survival, CP prevalence depends on the chosen definition of CP, the cut-off point regarding the level of motor impairment, health service organization, case findings and validation procedures. The same definition of CP is applied in CPUP as in earlier reports from Sweden and the SCPE [1,3,5,8,12-15]. Comprehensive and well organized child health services that are accepted by the population and free of charge, as well as the Swedish national registration number and population register facilitated the identification of children with CP. We believe that we have identified almost all children with CP in the area.

The number of children with CP born between 1990 and 1993 increased in 2002 compared to1998, in spite of the fact that three children had died. The prevalence in this cohort, now aged 8–11 years, changed from 2.4 to 2.8/1,000 (95% CI ± 0.4/1,000 in each). The main cause was high positive net migration. In the general population, the quotient of moving in and moving out of the region studied during the same four years was 1.6:1 compared to 23:1 in children with CP [22]. Efficient health care and child habilitation services may attract families of children with CP to the region and make them less likely to move away.

Every third child with CP who moved into the area was born abroad, compared to every twentieth child in the general population. The high prevalence of CP in children born outside Sweden could be due to a higher incidence of CP in their native countries. Another possible explanation, especially of the fourfold higher prevalence rate of children in GMFCS IV-V, is that more families with severely disabled children were allowed to remain in Sweden on humanitarian grounds. It is important to consider the more than double prevalence of CP among immigrant children and their more severe disability levels when planning health provision, schools and social services for children in communities with a high percentage of immigrants in Sweden. The severity of CP and the need for interpreters require more time and expertise on the part of the health services.

The total population approach is helpful in the planning of services. It also enables evaluation without selection bias.

In this study, the proportion of children whose impairment was classified as GMFCS level I, the mildest gross motor function limitation, was higher than in other CP-populations. In the present study GMFCS level I included 48% of children with CP born 1990–97 versus 32% in the cohort born 1991–98 in western Sweden [23]. Children who had received spasticity reducing treatment were excluded in the Canadian motor growth studies (28% GMFCS I) [24]. Data from the national CPUP database speak against significant bias in the ascertainment of CP as the cause of a high proportion of GMFCS I in this study. In children with CP living outside Skåne/Blekinge in 2006, the proportion of GMFCS I was 40% [unpublished data]. The detection of mild CP may vary between studies, as can the level of activity restriction required for a CP diagnosis. In order to avoid possible bias in the diagnosis of mild CP, the GMFCS specific prevalence rates can be used for comparison (Table 4).

In order to evaluate the results of the interventions and further refine the follow-up programme, it is necessary to divide the disparate CP-population into meaningful subgroups. The GMFCS, which was first introduced after the start of CPUP in 1994, has resulted in revisions to the programme. The recommendations concerning hip screening are now based on GMFCS levels instead of CP subtypes, also advocated by others [25]. In contrast to the CP subtypes, the GMFCS classification is valid and reliable and has been adopted worldwide.

There are many different versions of CP subtype classifications based on neurological symptoms and localization [3,26,27]. According to the Swedish classification, CP syndromes have different profiles in terms of functional abilities, general health, aetiology and timing of the brain damage [1,12,13,15-17,23,28-31]. The GMFCS classification alone is not a valid indicator of anything other than motor function abilities, even if the frequency and severity of associated impairments increase in line with higher GMFCS disability levels in group comparisons [13,28,29,32]. Accordingly, the total impairment load of children with CP correlates with the SCPE subclassification when combined with the GMFCS levels [23].

The GMFCS distribution in the different CP subtypes indicates similar use of the SC classification in our region and western Sweden [23,28]. Although the descriptions of neurological and other signs and symptoms were scrutinized by the authors in order to validate the subtype, some features probably remained undetected in this study. We found differences in the SC classification pertaining to dyskinetic syndromes. Guided by the American classification by Minear [26], some doctors used the term athetosis for any form of dyskinetic CP. Ataxic diplegia was not always recognized and severe spastic diplegia was sometimes classified as tetraplegia. In many cases sub classification had not previously been performed. The SCPE classification had not been used before, but was easy to become familiar with due to the fact that it is based on the SC.

Ataxic diplegia is rare and considered to be difficult to differentiate from spastic diplegia. Most epidemiological studies lump the two types of diplegia together, as recommended by Hagberg *et al.*[1,16]. Ataxic diplegia seemed to have a different gender and GMFCS distribution than spastic diplegia, which observation has, to our knowledge, not been previously reported or discussed. This may be a chance finding, but a similar gender distribution was found in a recent study from the north of Sweden [32]. In our experience, recognising ataxic diplegia and dysequilibrium syndrome as special subtypes within the SC ataxia group is helpful in the clinical setting [17,31]. For clinical purposes it is also important to separate spastic tetraplegia from other CP types, due to its specific functional profile in addition to motor dysfunction [16,33]. Professionals should be aware that reliability of the subclass classifications has not been established yet, even if judged meaningful in the clinical work. The correlations between motor development, other body functions and the clinical CP subtypes require more evaluation before a decision can be taken on which CP subtype classification, if any, should be used in the evaluation of different cerebral palsy treatment interventions and in the CPUP programme.

The main challenge of this follow-up programme is the early identification of children with CP and the maintenance of the database. As hip dislocation most often occurs between three and six years of age [9,34], all children under four years with any symptoms or signs of possible future CP should be included in the follow-up. This study showed that more than one third of the children with CP, included in the inventory for the first time, had not yet been offered participation in the CPUP programme. When invited to the CPUP follow-up, most parents of children with CP (98%) agreed to participate.

Systematic research is necessary in order to locate all children with CP. Even well-functioning child habilitation units may fail to detect the signs and symptoms in some children with CP, especially when other impairments such as mental retardation, autism, intractable epilepsy or different syndromes lead to more severe restrictions on activity.

## Conclusion

The prevalence of CP was influenced by migration/immigration and was 2.7/1,000 (95% CI 2.4–3.0), of which 1.4/1,000 (95% CI 1.2–1.6/1,000) were GMFCS-levels II-V. The subtype distribution and CP prevalence among children born in Sweden did not differ from that reported in other studies. To identify all children with CP and keep a CP register valid second opinion about classification of CP/CP subtypes must be available. Active search for undiagnosed CP among children with other impairments is necessary, both for the purpose of epidemiological research and to improve the quality of care.

## Abbreviations

BCSP – Bilateral Spastic Cerebral Palsy

CI – Confidence Interval

CP – Cerebral Palsy

CPUP – a clinical follow-up programme in combination with a health care quality database

GMFCS – Gross Motor Function Classification System

SC – the Swedish (Hagberg) Classification of CP subtypes

SCPE – the Surveillance of Cerebral Palsy in Europe network

USCP – Unilateral Spastic Cerebral Palsy

## Competing interests

The authors have no financial interest in this study, which was performed as part of the implementation and validation of the CPUP programme.

## Authors' contributions

The three authors initiated, developed and currently co-ordinate the further development of the CPUP programme.

LW and EN planned, performed and analyzed the results of the inventory. LW wrote and revised the manuscript with the active assistance of all the authors.

## Pre-publication history

The pre-publication history for this paper can be accessed here:


